# Indigenous farmers’ perceptions of problems in the rice field agroecosystems in the upper Baram, Malaysia

**DOI:** 10.1186/s13002-022-00511-1

**Published:** 2022-03-29

**Authors:** Alexander Hollaus, Christoph Schunko, Rainer Weisshaidinger, Poline Bala, Christian R. Vogl

**Affiliations:** 1 University of Natural Resources and Life Sciences, Vienna, Department of Sustainable Agricultural Systems, Division of Organic Farming, Gregor-Mendel-Strasse 33, 1180 Vienna, Austria; 2 Agroecology.AT, Consultancy on Agroecology and Sustainability of Agricultural Systems, Hauptstrasse 22, 2120 Obersdorf, Austria; 3 Institute of Borneo Studies, Universiti Malaysia Sarawak (UNIMAS), 94300 Kota Samarahan, Sarawak Malaysia

**Keywords:** Indigenous and local knowledge, Landscape ethnoecology, Spatial perceptions, Indigenous agroecology, Rice field, Landscape management, Borneo

## Abstract

**Background:**

Rice field agroecosystems produce food for more than half of the world’s population and deliver important services supporting farmers’ livelihoods. However, traditional rice field agroecosystems are facing a variety of problems, including pests or markets that are hard to access. This research explored indigenous farmers’ perceptions of the problems, their causes and consequences, and the solutions applied to address them in the rice field agroecosystem. Furthermore, the study investigated how indigenous farmers related these problems to the surrounding landscape elements and to microzones in the fields.

**Methods:**

Data were collected in two villages in the upper Baram, Sarawak using a qualitative approach that included sketch drawings and face-to-face interviews. Forty-three indigenous farmers of the Kenyah, Penan and Sa’ban ethnic groups were interviewed in their rice fields. The sketch drawings were used to identify the perceived landscape elements, while the oral interviews were employed to identify perceived microzones. Furthermore, the interviews elicited the perceived problems in the rice field agroecosystem and their relations to landscape elements and microzones.

**Results:**

The findings identified a total of nine environmental problems, e.g. animal disturbance, six social problems, e.g. difficult to access farm inputs, and eight agricultural technology system problems, e.g. poor soil quality, with some found to be rooted in complex causes and affecting agricultural productivity. While some problems were perceived at field level, microzones were frequently used as sub-field indicators of the problems. The surrounding landscape elements were perceived as both a source of the problems and as a means of avoiding them. To solve the problems, farmers applied preventive and reactive strategies based on traditional knowledge and scientific knowledge, resulting in a hybridisation of knowledge systems.

**Conclusions:**

By including environmental, social, agricultural technology system problems and different spatial scales, this research contributes to addressing issues that can be overlooked when focusing on only one dimension of the problems. These results contribute to a better understanding of how indigenous farmers perceive, cope with and adapt to problems in rice field agroecosystems, which is important for landscape management.

**Supplementary Information:**

The online version contains supplementary material available at 10.1186/s13002-022-00511-1.

## Background

Rice field agroecosystems (RAEs) are important man-made ecosystems that produce rice (*Oryza sativa*) as a staple food for more than half of the world’s population and support farmers’ livelihoods through subsistence, income and cultural services [1, 2]. Farmers grow rice in a variety of agroecosystems, depending on their environmental and social settings and on the agricultural technology systems applied [3]. The International Rice Research Institute (IRRI) divides RAEs into four broad categories: rainfed upland rice, irrigated rice, rainfed lowland rice and flood-prone rice systems [4, 5]. In RAEs cultivated by indigenous farmers, the management is based on a wide body of traditional knowledge and perceptions [6–10] that have been gained over a long period of continuous practice and interaction with the surrounding environment [11]. The rice terraces of Banaue, Philippines, for example, are an irrigated RAE with a sophisticated irrigation system built and traditionally managed for generations by indigenous people [12, 13]. Another RAE that has long been rooted in the sociocultural life of indigenous peoples is rainfed upland rice, which is often grown by means of shifting cultivation techniques [14, 15]. Owing to their lengthy experience of the surrounding environment, indigenous farmers’ traditional knowledge is rich in locally adapted information about environmental conditions that are important for landscape management, decision-making and problem-solving [16–18]. Porter-Bolland et al. (2012), for example, show that community-managed forests, whose management is largely based on traditional knowledge, are similarly effective at reducing deforestation as areas under protected status [19]. However, indigenous farmers’ traditional knowledge is dynamic and adaptive, and is influenced by historical, cultural and environmental factors, as well as through interaction with scientific knowledge systems [10, 11, 20, 21]. As is suggested for the Tsimane’ indigenous community in Bolivia, their perception and use of the landscape may have changed as a result of their recent integration in the market economy and opening up to outsiders [16]. A change in the environmental settings, social settings and agricultural technology system of traditional managed RAEs requires indigenous farmers to adjust to new conditions, such as adopting new farming practices or engaging in the market economy, which can cause problems in the RAEs and with farmers’ livelihoods [7, 12, 14, 21, 22]. Some of the problems faced by farmers in RAEs are issues related to invasive as well as native pests [12], water shortage issues, market access difficulties [23], pesticide-related health problems [24] and the abandonment of rice fields due to farmers’ outmigration in search of employment [22]. Problems impacting traditional rice farming systems, including those classified by the FAO as Globally Important Agricultural Heritage Systems (GIAHS)[25], may be seen in Japan, where youth outmigration has resulted in the discontinuity of traditional practices and land abandonment [26], in the Hani rice terraces in China, where extreme weather events, steep slopes and abandonment of farmland contribute to landslide problems [27] and in the Philippines’ Kiangan rice terrace system, where difficulties originate from the introduction of invasive pests into rice fields and from unregulated land conversion resulting in soil erosion and water supply problems [28].

In Sarawak, Malaysia, RAEs are impacted by problems related to youth outmigration [14, 29–33], loss of traditional rice farming knowledge [14, 34] and the expansion of plantations, infrastructures and industrial logging [8, 35–37]. Some of the land and labour that would have been available for rice farming has instead been diverted to logging, plantation, cash crop planting and tourist operations [9, 30, 36]. Aside from the socioeconomic issues, agricultural technology system problems caused by poorly levelled wet rice ponds can be the result of a lack of animal or machinery power and lead to uneven water distribution in the field [38]. Other problems are known to be associated with upland rice, an RAE that is widely found in the uplands of Sarawak, and are caused by agricultural intensification and the shortening of fallow periods. Both are factors that can contribute to environmental issues that are linked to a decrease in fertility and degradation of the soil [9, 39]. In addition to soil-related issues, indigenous farmers in the interior of north Sarawak perceive problems in rice cultivation related to climate change issues such as droughts, floods and poor years for agriculture in general [40].

The environmental, social and agricultural technology system problems do not just occur on field level or spatially closed RAEs, but also manifest themselves in spatial sub-units within the rice fields and are impacted by the surrounding landscape. The landscape is categorised by indigenous farmers into landscape elements, often based on the principal domains of biotic and abiotic criteria, human interventions, and their potential uses and functions [16, 17]. The landscape is therefore “an arrangement of biotic, abiotic and cultural landscape elements recognised and referred to by common nouns (generic landscape terms or categories), rather than proper nouns (place names or toponyms)” [41]. In general, landscape configuration and composition affect the ecological processes that occur in landscapes and landscape elements, such as hydrological flows and variation within catchments [42] or on the RAE’s arthropod population [43]. According to Ali et al. (2020), the landscape around rice fields influences the insect pest population, indicating that landscape structure should be considered when implementing integrated pest management [44]. Similar effects have been reported in Sungai Semanok, Sarawak, where rice farmers lack access to the same high-quality pesticides as nearby oil palm plantations, resulting in pest migration to rice fields and thus negatively affecting agricultural productivity [34].

On a smaller scale, farmers perceive ecological and agronomic processes in spatial sub-units within landscape elements that are addressed by the practices they apply [45]. These spatial sub-units or microzones are patches of homogeneous characteristics perceived and articulated by farmers [45, 46]. For example, Mongolian farmers perceive microhabitats such as vegetation that has higher nutrient needs on marmot burrows or weeds along fences or around manure heaps [47], while the Tsimane’ divide their landscape sub-units based on the dominance of plant species [16].

**Fig. 1:**
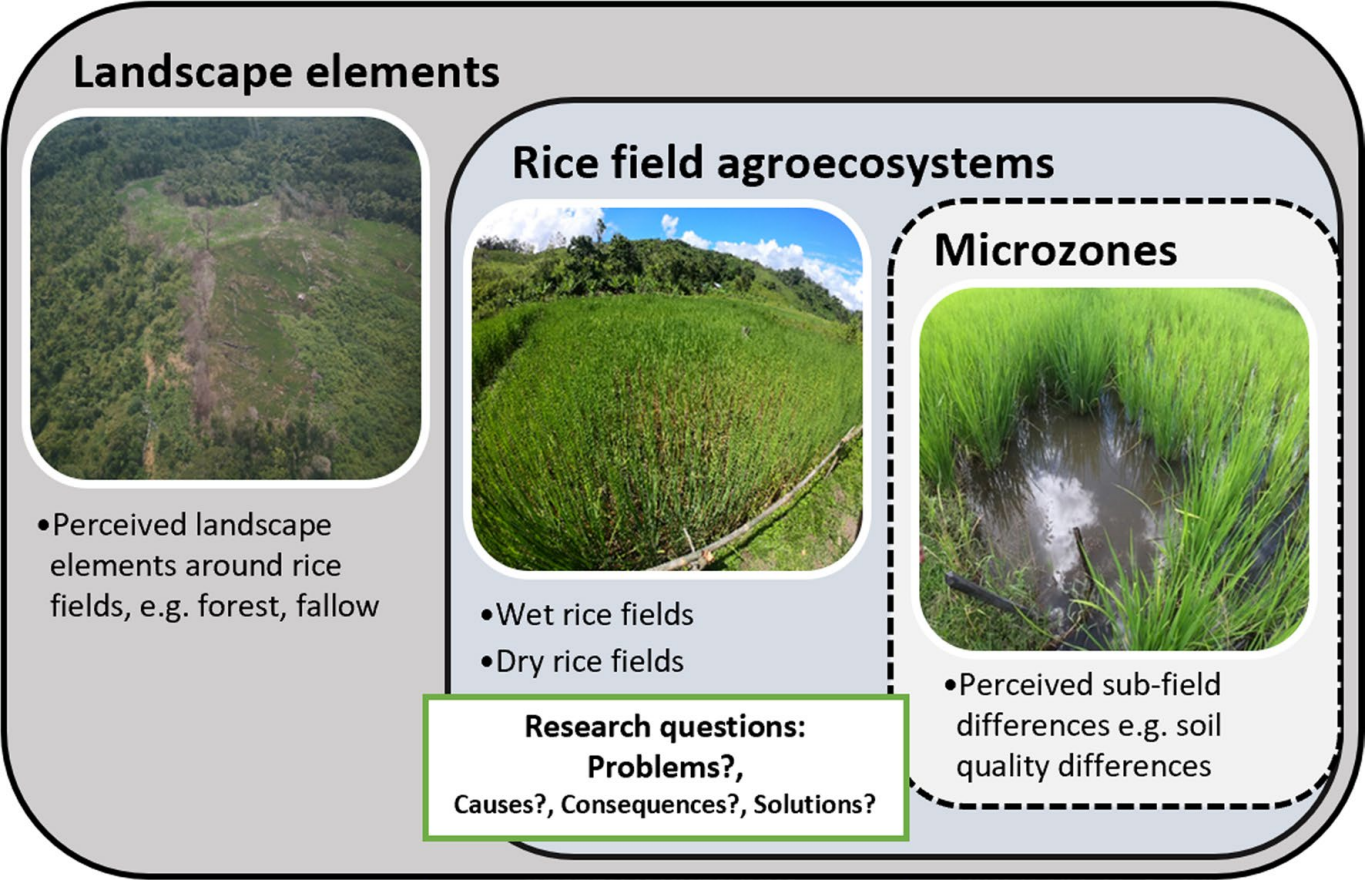
Spatial scales of analysis to identify problems farmers perceived in the rice field agroecosystem (Photo: A. Hollaus)

The purpose of this study was to examine indigenous farmers’ perceptions of problems in the RAEs in two villages in Sarawak’s upper Baram region. Our research questions were as follows: (i) What problems do indigenous farmers perceive in their RAE? What are the underlying causes and consequences of these problems? What solutions do farmers apply to address these problems? In this study we also went beyond the field level as an analytical spatial unit and examined (ii) how problems are related to microzones in the rice fields and landscape elements surrounding the rice fields (Fig. 1). We examined the problems in the RAEs of dry and wet rice fields found in the upper Baram. Rainfed dry rice fields are cultivated using a shifting cultivation technique, while wet rice fields remain in their location and are situated in naturally swampy areas or are flooded by a water source, such as by irrigation or rainwater.

A landscape ethnoecological approach was chosen to undertake this study. Landscape ethnoecology examines how local people perceive, identify and manage their landscape [48] and “how human societies conceptualize the environments on which they depend.” [16]. Many landscape ethnoecological studies concentrate on the emic classification of landscapes, including comparisons with scientific classification, and their meaning, value, usage and management by indigenous and local people [16, 41, 49–52]. However, this study did not focus on a complete list of landscape elements or microzones perceived by indigenous people, but rather on using landscape elements and microzones as spatial analytical units to identify indigenous farmers’ perceived problems in the rice fields. Given the significance of RAEs to indigenous farmers in the upper Baram, we anticipate that the study’s findings will contribute to a better understanding of local challenges facing indigenous farmers, as well as their problem-solving strategies.

## Methods

### Study area

**Fig. 2:**
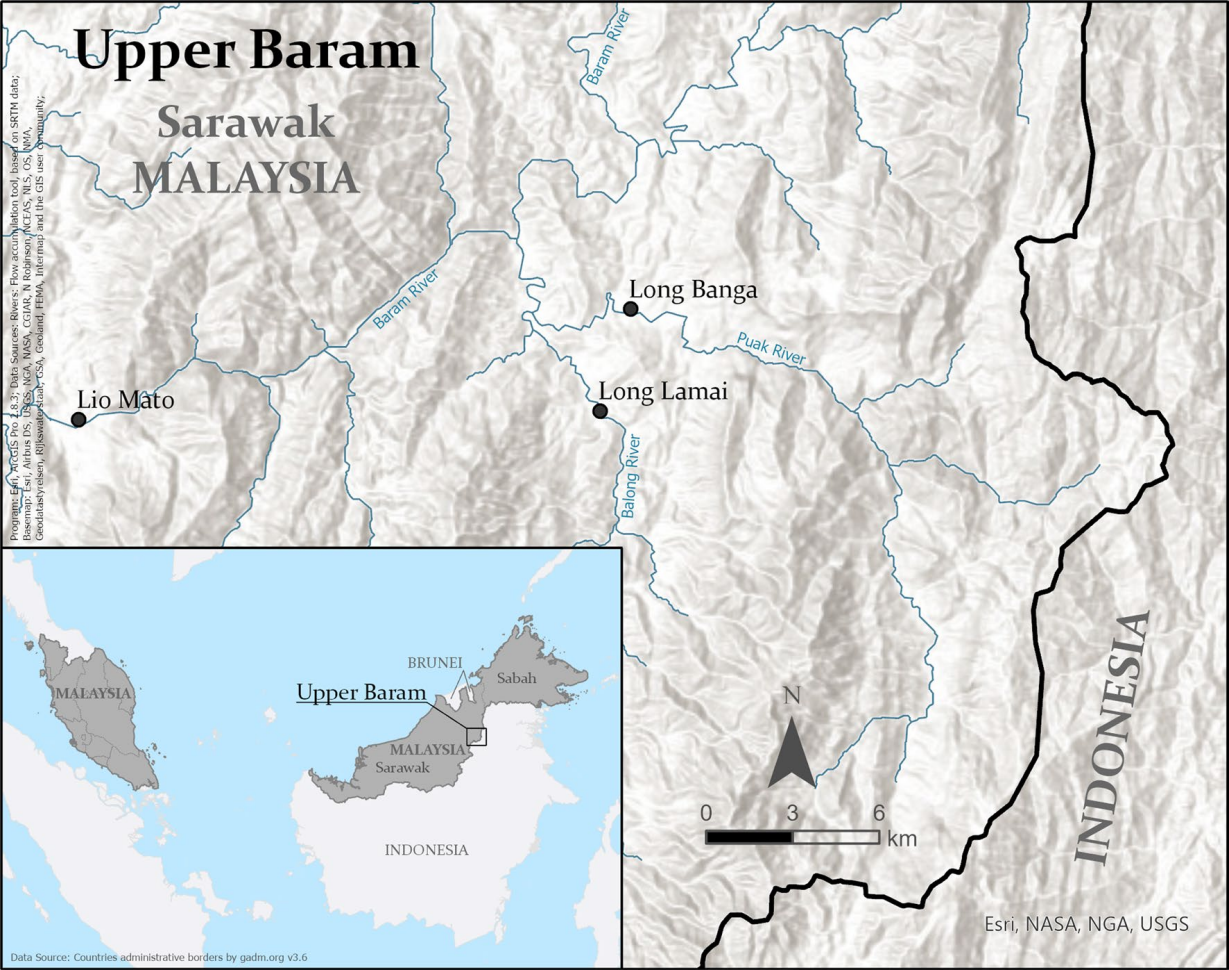
Location of the upper Baram region in Sarawak, Malaysia, with the two research villages of Long Banga and Long Lamai

We conducted research in the upper Baram region (Fig. 2), in Sarawak, Malaysia, which is a landscape that has a multi-ethnic population. The upper Baram region is situated in the interior uplands of Borneo island, along the upper reaches of the Baram river. The tropical climate is characterised by a limited expression of seasonality, with a dryer period during the SW monsoon in May to September and a wetter period during the NE monsoon from November to March, mean annual precipitation of 3352 mm for Lio Mato [53] and an average temperature of 27.8 °C (derived from WorldClim.org v2 data for Long Banga [54]). The hilly area is covered by a mixed dipterocarp rainforest [55]. Dry and wet rice fields are found around the villages, as well as landscape elements that are related to the RAEs. Rice fields are cultivated by the indigenous farmers with a number of traditional but also imported dry and wet rice cultivars, including the dry rice varieties locally known as *Padi Turi* and *Padi Pulut* as well as wet rice varieties of *Padi Adan* and *Padi Kanowit*. The varieties from the two distinct rice field agroecosystems are important as they offer a variety of flavours and are used by the indigenous farmers for several different purposes.

The research focused on two villages, Long Lamai (3°10′20"N, 115°23′7"E) and Long Banga (3°12′13"N, 115°23′36"E), in the upper Baram, which are approximately 4 km apart and at an altitude of approximately 440 m ASL (SRTM derived data [56]). Access to Long Banga is by a small airstrip and a logging road that connects the village to the coastal area. Long Lamai can only be accessed by a long boat or after a two-hour hike from Long Banga. The villages are inhabited by three ethnic groups: Penan, Kenyah, and Sa’ban. Long Lamai village, which has about 500 inhabitants, is home to the Penan ethnic group [33]. In Long Banga approximately 350 Sa’ban and 230 Kenyah (personal communication with the contact person in Long Banga^1^), most of them belonging to the sub-group of Kenyah Lepo Ke’, live in the village.

The three ethnic groups are usually referred to as *Orang Ulu*, which translates as “those who dwell upstream or in the interior”, an expression that encompasses several ethnic groups with different languages and cultures residing in Sarawak’s uplands [57, 58]. The Kenyah and Sa’ban are traditionally agriculturalists and migrated to the upper Baram region in the twentieth century when the Penan were already in the area [59, 60]. The Kenyah traditionally resided in longhouses with verandas, where traditional festivals were celebrated, and rice cultivation is an important cultural element as well as essential for people’s livelihoods [8, 61, 62]. While the societies of the Kenyah and Sa’ban were stratified [62], the Penan, who were once nomadic hunter-gatherers, are described as an egalitarian organised group and are distinguished by their language dialects into Western Penan and Eastern Penan [63, 64]. Most of the Penan have become sedentary and only a small number of Penan continue to live in the tropical forest as nomadic hunter-gatherers [60]. In Long Lamai, the Eastern Penan have started to settle and farm in the 1950s [59, 60]. However, hunting and gathering in the forest has continued to be an important element of the livelihoods [61, 63]. Nowadays, Christianity has increasingly displaced animism as the predominant religious belief system among all three ethnic groups [59, 63]. However, continued industrialisation, modernisation and external pressures are further influencing indigenous peoples’ traditional ways of life [60, 63].

### Sample

The research population consisted of indigenous farmers who cultivate wet or dry rice fields in the two villages of Long Banga and Long Lamai because they are the traditional knowledge-holders for managing and organising the local RAEs. These villages were selected because they are representative of the region’s diverse ethnic groups and infrastructure contexts, yet share a similar natural setting due to their proximity.

We used a snowball sampling strategy [65] to identify participants and asked the two contact persons in each village, both of whom are rice farmers, for potential participants. The participants needed to meet the criterion of being a rice farmer in one of the two villages. These participants were then asked to identify additional informants for the study. Interviews were held with a total of 43 participants and covered 50 rice fields because some participants were questioned about both dry (27) and wet (23) rice fields (Table 1). The mean age of the participants was 52.85 years (min. 24, max. 76) and included 15 females and 28 males of the three ethnic groups (12 Kenyah, 15 Penan and 16 Sa’ban).

**Table 1:** Overview of interview participants of each ethnic group

Variables	Ethnic group			Sum
	Kenyah	Penan	Sa’ban	
Female participants	4	5	6	15
Male participants	8	10	10	28
Sum participants	12	15	16	43
Dry rice field interviews	10	8	9	27
Wet rice field interviews	2	11	10	23
Sum rice field interviews	12	19	19	50

### Data collection

#### Qualitative interviews

Data were collected by the first author during a three-month field trip to Long Lamai and Long Banga in early 2020. Before conducting the qualitative interviews, the village leaders were asked for permission to conduct research in the villages, signing a letter of Free, Prior and Informant Consent. Furthermore, each interviewee was first asked to participate and to sign a written letter of consent for the data to be used. The letters were provided in English and Malay. The interviews were conducted in English with English-speaking people (n = 14). If an interview could not be held in English (n = 29), the interview was translated in situ by a local interpreter from the interviewee’s language (Penan, Sa’ban, Kenyah) or Malay into English.

**Fig. 3:**
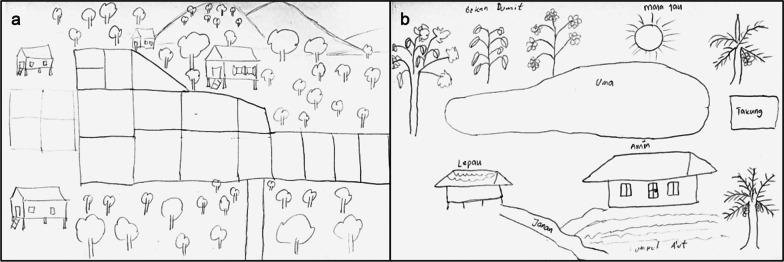
Examples of sketches drawn by farmers. **a**: wet rice field (WR, Female, Sa’ban, 24); **b**: dry rice field (DR, Female, Kenyah, 42)

Data were gathered using participatory sketch drawings (Fig. 3) in combination with semi-structured interviews. Sketch drawings are effective for capturing spatial knowledge and perceptions of the environment [66–69]. While participatory sketch drawings are effective at establishing spatial relations and representation [66, 67], without an additional language-based approach, they may lead to the omission of important language categories [67]. Therefore, we combined the sketch drawings with oral interviews to understand problems connected to landscape elements.

The sketches were drawn after providing the interviewees with a B5-size sketch book and asking them to draw their rice field. To include the surrounding area in the sketch and stimulate the drawing process, we followed up by asking what was around the rice field. Besides these two guiding questions, the participants were given free expression in their drawings and were not subjected to time constraints or other restrictions. However, if the participants just answered verbally, we invited them to draw the feature they had mentioned in the sketch. Eleven interviewees declined to draw themselves and instead instructed the interpreter on what to draw. After the participants confirmed that the drawing was complete, the relations between the rice field and the landscape elements depicted on the paper were addressed by asking farmers whether it was good or bad that the landscape element was next to their rice field and why, and whether the landscape element caused any problems. Microzones were identified by asking the farmers about perceived differences within the rice fields and why these differences occurred. To initiate a discussion of problems at field level, the farmers were asked to describe the RAE characteristics and the differences from other RAEs. By adopting this approach, it was possible in the interviews to explore the problems, their causes and consequences, as well as the solutions applied by farmers. The interviews were recorded and subsequently transcribed verbatim. For data analysis, the sketch drawings were digitised.

### Data analysis

The first author conducted a qualitative content analysis of the drawings and transcripts by data-driven (inductive) and concept-driven (deductive) coding [70]. Two conceptual steps in the analysis were performed to answer our research questions in order to: (i) identify the landscape elements and microzones, and (ii) identify all the problems and their causes, consequences and solutions applied, and connect the problems to the landscape elements and microzones.

The microzones were inductively coded by identifying features at sub-field scale perceived by farmers as having different properties throughout the rice fields and as being associated with the problems. The landscape elements were derived from the inductive analysis of the sketch drawings and transcriptions concerning features around the rice field that were drawn or spoken about. The 44 features obtained from the sketch drawings and oral interviews were categorised into 16 landscape elements [see Additional file 1].

To perform a detailed analysis of the problems, we applied the same conceptual framework as in the interviews (problem, causes, consequences and solutions) to guide the coding. Thus, we coded all the problems mentioned in the interviews, their causes, consequences and applied solutions, and assigned the problems according to their primary cause into one of the three agroecosystem settings: environmental problems, social problems, and agricultural technology system problems [3]. If a problem was derived from or linked to a landscape element or microzone, a code was applied to link the spatial unit to the problem. In the results section, we present the findings in a table that links the problems to the causes, consequences and solutions, and to the landscape elements and microzones. Furthermore, the findings were supported by quotations from the transcriptions. We have not changed the verbatim quotations, but provide omissions, additions and translations in square brackets for a clearer understanding of the farmers’ statements. The information about the participant who provided the quotation states whether it was from a dry rice (DR) or wet rice (WR) field interview, and the participant’s ethnic affiliation, gender and age. Quotations that were translated in situ by an interpreter are marked by “VI”. The qualitative data collected from the sketch drawings and the transcription of the audio recordings were analysed using the MAXQDA program [71].

## Results

### Microzones

**Fig. 4:**
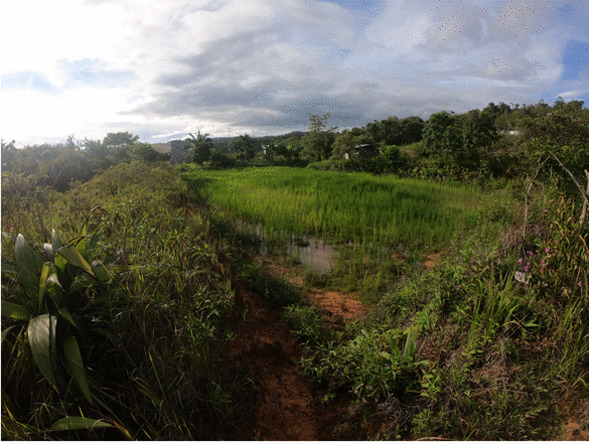
Wet rice field showing microzones of patches of poor plant growth (yellowish rice plant colour), soil colour difference (reddish and black soil colour), water level differences (areas with and without water) and field level differences (higher and lower areas). The rice field in the picture was levelled using a bulldozer, often seen as a problem due to the red infertile soil beneath the fertile black topsoil being uncovered (Photo: A. Hollaus)

**Fig. 5:**
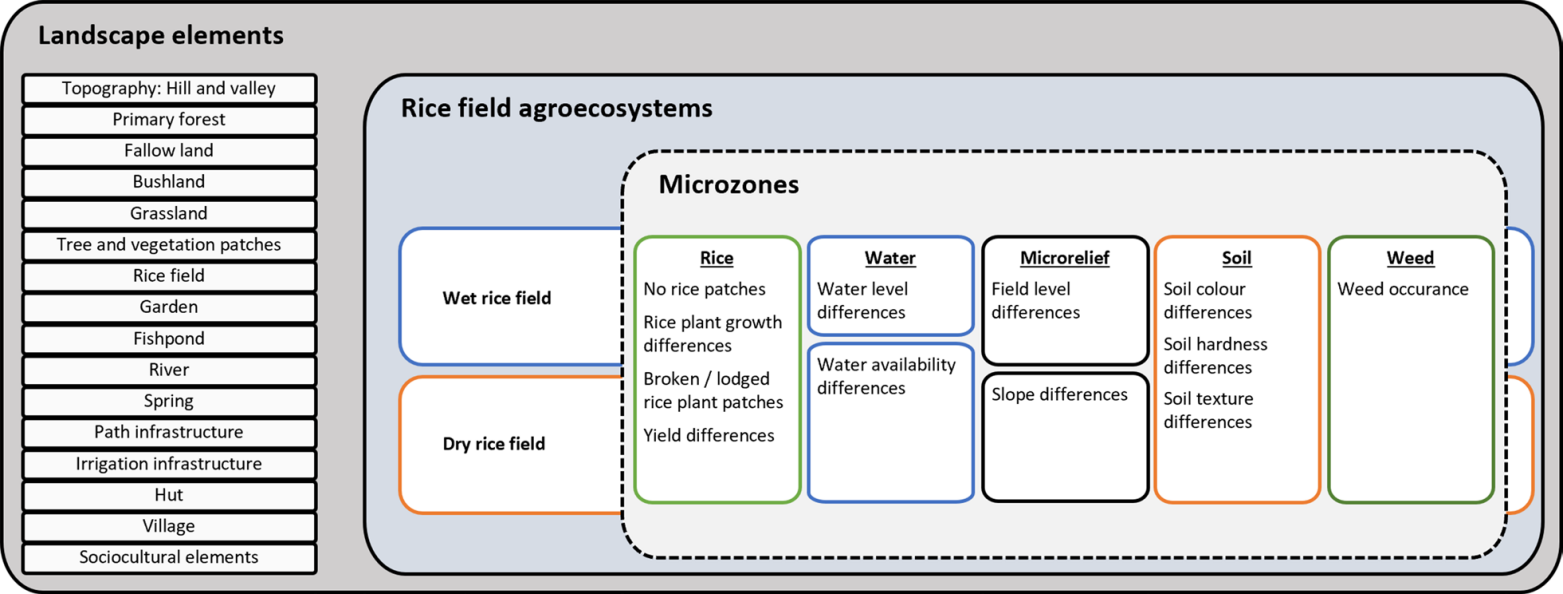
Overview of landscape elements, rice field agroecosystems and microzones in the rice fields

In the rice fields, we found that farmers perceived microzones (Fig. 4) in terms of five features: rice plants, water, microrelief, soil and weeds (Fig. 5). The microzones formed by rice plants are linked to indicators of broken or lodged rice plant patches, no rice plant patches, rice yield differences and rice plant growth differences. To identify problems with rice plant growth, the farmers used the properties of plant colour, plant growing, tiller quantity, yield and grain quality (Table 2).

**Table 2:** Indicators mentioned by farmers for assessing rice plant growth

Variables	Healthy	Unhealthy
Colour	Green	Yellow, black, brown (spots)
Tiller	Many	Few
Grains	Large, full	Small, empty, fewer seeds
Yield	High	Low
Growing	Tall, fast	Small, slow

Other microzones were perceived by soil properties based on texture, hardness and colour. In dry rice fields, farmers also noticed microzones in the microrelief by slope differences. In wet rice fields, microrelief microzones were perceived in terms of field level differences of small depressions or elevations. Furthermore, differences in the wet rice field were recognised by water level differences and by different water availability in wet rice and dry rice fields. Furthermore, weed occurrences in the field formed microzones that were recognised by farmers and led to problems in the RAEs.

### Landscape elements

**Fig. 6:**
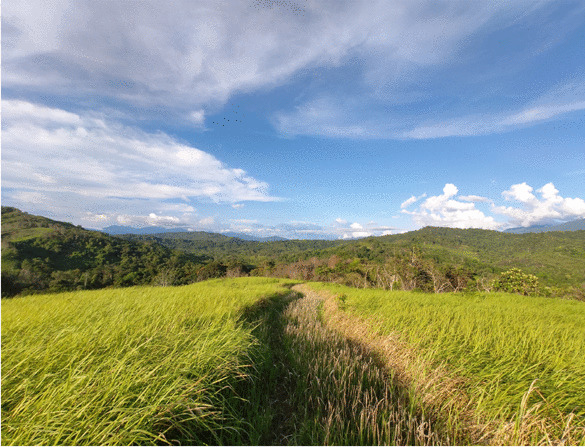
In the foreground, the landscape element of grassland dominated by *Imperata* *cylindrica*; in the background, a patchy pattern of dry rice fields and fallow land on hills (Photo: A. Hollaus)

The landscape surrounding the rice fields was composed of topographic elements (hills and valleys), natural ecosystems (primary forests, tree and vegetation patches, rivers, springs) and man-made or semi-natural ecosystems (fallow land, rice fields, gardens, bushland, grassland, fishponds), as well as artificial elements (path infrastructure, huts, villages, irrigation infrastructure) and sociocultural elements (borders, ancient graves, historical places) (Fig. 5). In the conception of indigenous farmers, land covers changed from unfarmed (e.g. primary forest) to farmed (e.g. rice field) and fallow land (e.g. secondary forest). Consequently, fallow land was the landscape element around rice fields mentioned most frequently, suggesting a highly farmed area. Fallow land can range from grassland to forest, encompassing the landscape elements of bushland and grassland if they used to be cultivated (Fig. 6). Since some of the farmers attributed certain weed-related problems to these landscape elements, we separated them from fallow land. Adjacent rice fields, which in some locations are clustered together, were frequently referred to as a landscape element. Another man-made ecosystem often mentioned was gardens, which are seen as an important component of the dry rice fields. As one farmer put it:

Always Sa’ban and Kenyah if they do the dry paddy [rice] field, they have to make small era [garden] near the [rice field]... (DR, Male, Sa’ban, 58)

Topographic elements were mentioned by participants in the form of valleys and hills. An artificial element in the landscape was huts that are used to store rice, equipment and materials, as well as provide shelter for relaxing or remaining in the rice field. Huts ranged in quality from a few branches to pillars and planks mainly made from local materials. Besides the artificial element of a hut, path infrastructures were important for travelling to the rice field and ranged from simple walking paths through the rainforest to concrete roads. Other artificial elements in the landscape were villages, including houses as well as the airfield in Long Banga. Furthermore, an important artificial landscape element was the irrigation infrastructure, which included pipes and dams, but also a micro-hydropower plant whose discharge water was used to irrigate wet rice fields. Water was also obtained from natural ecosystems such as rivers and springs, including salt springs. Another natural ecosystem was primary forests, which were referred to as already being a long way from the villages but with which farmers associated fertile soil. Sociocultural elements referred to by the farmers were historical village sites, borders and ancient graves.

### Problems, causes, consequences and solutions in rice field agroecosystems

In the interviews, 23 problems were identified in the RAEs, of which nine, six and eight were categorised by their primary cause as environmental, social or agricultural technology system problems, respectively (Table 3).

**Table 3:** Indigenous farmers’ perceived problems, causes, consequences and solutions, as well as associated landscape elements and microzones

Problem		Causes	Consequences	Solutions	Landscape elements		Microzones
					Problem sources	Problem mitigation	
***Environmental problems***							
*Animal problems*	Animal attack	Field location	Health risk	Greater awareness			
	Animal disturbance	Field location Individual divergent planting times	Poor rice plant growth Yield loss	Coordinated planting time Cultivate both types of rice fields Scarecrow Clearings of vegetation around the field Fencing Net Trapping Not planting certain crops Hunting Guarding Draining Hand-picking Pesticides	Fallow land Village Bushland Path infrastructure Primary forest Hill Garden Rice field	Rice field Path infrastructure Village Hut	Broken/lodged rice plant patches No rice plant patches Poor rice plant growth patches
Steep slope in dry rice fields		Land availability	Affect the amount of yield/area Health risk Difficult work Erosion	Avoid steep areas	Hill		Slope differences
*Vegetation problems*	Falling branches and shade from vegetation	Surrounding vegetation	Poor rice plant growth	Clearing	Fallow land Garden Hill Tree/vegetation patches		
	Weed occurrence	Weed migration Poor soil quality Escape of fire Insufficient water availability Unadjusted water level Uneven levelled field No weeding related to not having time and health Erosion	Poor rice plant growth Weed resistance Poor soil quality Health risk Difficult work	Burning of weeds Avoid weed-dominated areas Synthetic herbicides Weeding by hand, knife or machine Clearings of vegetation around the field Retain water in the field Wait for shade by natural vegetation succession	Bushland Grassland Fallow land Rice field	Fallow land	Poor rice plant growth patches Weed occurrence Water availability differences Water level differences Field level differences
*Weather problems*	Drought	Late/early planting time Poor season	Poor rice plant growth	Store enough rice for several years			
	Hot temperature	Late/early planting time Poor season	Poor rice plant growth Health risk	Rest in shadow		Fallow land Tree/vegetation patches	
	Strong wind		Lodging	Drain rice field Make rope to support rice plants			Lodged rice plant patches
	Too much rain	Late/early planting time Poor season	Poor rice plant growth Slippery condition Flooding	Stay at rice field	River		
**Social problems**							
Human disturbance		Step on rice plant Fishing	Poor rice plant growth Broken rice plants	Fencing The owner takes fish him/herself	Path infrastructure River Rice field Tree/vegetation patches		
Hard to afford and lack of access to paid labour, machinery, farm inputs, tools or irrigation infrastructure		No income/job No support from authorities or NGO	Do not have tools, irrigation infrastructure, farm inputs, machines, paid labour More manual work Smaller rice field Only dry rice field cultivation	Ask others to help in the rice field Neglect tasks in rice field Government supply Gradual expansion of wet rice field NGO supply Go working to earn money	Irrigation infrastructure		
Other tasks/jobs besides farming		Need money to support the family Need money to buy farm input or tools or paid labour	Less time for rice field Weed occurrence	Neglect rice field			Weed occurrence
Poor access to rice fields		Poor infrastructure condition Far distance of the field Poor health of the farmer	Difficult to reach Don’t cultivate the land Path in poor condition cannot use vehicle	Borrow land Use land next to path infrastructure or close to the village	Path infrastructure	Path infrastructure Village River	
Poor capability of farmers		Poor health or high age of the farmer	Limitations in conducting tasks in the field Stop cultivating dry rice field Difficult to access the rice field	Help from others Paid labour/group work Field close to the village		Village	
Taboos		Belief and religion	Sickness if area used	Avoid area	Sociocultural elements		
**Agricultural technology system problems**							
Spread of fire		Poor management of burning Weather during burning	Loss of valuable trees Unintentionally burnt area Weed occurrence	Inform neighbours Burning time/method	Garden Tree/vegetation patches		
Insufficient water availability		Broken irrigation Infrastructure Lack of irrigation infrastructure Poor irrigation organisation Uneven levelled field Poor weather season Poor soil quality	Poor rice plant growth Only dry rice cultivation Weed occurrence Hard soil quality	Maintain irrigation infrastructure Use excess water from another rice field Abandon field/plot Levelling Buffer strip above field Rice variety adaption Weed management	Grassland Rice field Hill Irrigation infrastructure River	Fallow land Fishpond Rice field River Spring Irrigation infrastructure Valley	Yield differences Poor rice plant growth patches Water level differences Water availability differences Field level differences Slope differences Soil texture differences
Lack of suitable land		Poor land quality Suitable land owned	Limitation in the use of the borrowed field Location of field Only cultivate dry rice field Use poor land quality Land occupation and land conflict	Borrow land Maintain ownership of land Avoid using poor quality land Tolerance to cultivate own field by others	Fallow land Bushland Grassland	Fallow land Valley	
Insufficient rice yield		Bad agricultural year		Store enough rice for several years Planting both rice field agroecosystems Help and exchange Buy rice			
Poor soil quality		Insufficient water availability Overuse of field Fallow vegetation and length Topographic location of the field Erosion Poor levelling	Poor rice plant growth Less yield Weed occurrence Affect usage frequency Difficult work	Fertiliser application Change the size of the rice field Find suitable soil/shifting Maintain longer fallow period Keep water in the rice field Buffer strip above field Return fertile soil	Fallow land Grassland Bushland Hill Path infrastructure River	Fallow land Primary forest Valley Garden	No rice plant patches Poor rice plant growth patches Yield differences Water availability Field level differences Slope differences Soil colour, hardness, texture differences
Synthetic fertiliser application		Poor soil conditions	Lodging Poor rice grain quality	Avoid application Buffer strip above field Mulching in wet rice field			Lodged rice plant patches Soil quality differences Poor rice plant growth patches
Synthetic pesticide application		Weed occurrence Animal disturbance	Poor rice plant growth No effectiveness Water pollution Health risk Lower selling price	Avoid application Weeding	River Fishpond		Weed occurrence
Unadjusted water level in wet rice fields		Poor water management	Harvest risk of wet grain Difficult work Lodging Drown rice plant Weed occurrence	Blowing water out of rice field before harvest			Lodged rice plant patches Water level differences

### Environmental problems

#### Animal problems

Animal attacks and animal disturbance were the environmental problems to which the farmers referred. Animal attacks were mentioned by one farmer who stated that snakes and bears posed a health risk for farmers in rice fields further away from human activities, and required greater awareness.

In contrast, animal disturbances were significant problems for the RAEs that were frequently mentioned in the interviews. In the rice fields, animals eat or damage rice plants, thus having a detrimental impact on rice plant growth and yields. Animal disturbances were linked to rice fields further away from human activity and to individual divergent planting times. The pests most frequently mentioned in the interviews were the sambar deer (*Cervus unicolor*) and bearded pig (*Sus barbatus*) and birds. Farmers also mentioned monkeys, including pig-tailed (*Macaca nemestrina*) or long-tailed macaques (*Macaca fascicularis*), which invade rice fields and destroy the rice plants.

They [monkeys] roll over the paddy [rice plant] and the paddy is stuck in [the fur]. […]. Yeah, and then, later, on the high tree and they take [and eat the rice]. (DR, Female, Sa’ban, 57)

Other animals were wild birds and chickens that were blamed for eating the rice seeds or trampling on the young rice plants in search of worms. Furthermore, farmers mentioned various insects, such as worms, caterpillars, grasshoppers, locusts and beetles as the cause of poor rice plant growth. A relatively recent phenomenon affecting rice plant growth is the golden apple snail (*Pomacea* sp.), which was introduced from an unknown source and is spreading over wet rice fields.

Farmers used a variety of methods to minimise animal disturbances. One method was to plant both types of RAEs as a precautionary step in the event that one type of rice crop fails to yield. A farmer explained why:

[...] it’s for the safety. [...] Because payau [sambar deer] come and eat the parai [rice] at the terek [dry rice field] and then they still have the taka [wet rice field] to cover [their food needs]. (DR, Male, Penan, 35, VI)

Furthermore, farmers suggested that one way to spread the risk of animal disturbance was to adhere to the community planting time, which is coordinated between the villages. Observing the community planting time and pesticide spraying were the strategies mentioned to control insect pests. Scarecrows, generally upright poles with a human scent (e.g. t-shirts) or with noisy objects (e.g. cans) hanging from them, were another possibility for minimising animal disturbances. Similarly, walking along a clearing of vegetation around the field spreads human scents that should keep animals away. Additionally, fences made of rattan or vine plant ropes are an easy way to prevent sambar deer in particular from entering the rice field. Other methods mentioned for controlling animal disturbances included installing a net to capture birds, guarding the rice field, setting animal traps and hunting for animals. Some farmers mentioned avoiding planting crops that attract animals. Attempts had been made to remove the golden apple snail, which is a problem only in wet rice fields, by draining the water. However, this method could lead to the establishment of weeds, which are also harmful to rice plant growth. Thus, farmers reported attempting to collect snails by hand or using pesticides.

Microzones indicating animal disturbance were patches of poor rice plant growth, patches of broken rice plants or patches with no rice plants. For example, one farmer explained that a patch with no rice plants was caused “*because of payau [sambar deer]” (WR, Male, Penan, 41, VI).*

Landscape elements related to animal disturbances were fallow land, bushland, primary forest, hills, other rice fields, villages and path infrastructures that act as a habitat for animals and therefore as a source of the problem. Gardens also contributed to the problem by attracting animals with the crops planted there. To reduce animal disturbance, farmers favoured having their rice fields close to a path infrastructure, especially the main path, or adjacent to huts, villages or other rice fields since the presence of humans was perceived to reduce animals in the field.

#### Steep slopes

In dry rice fields, a steep slope poses the risk of falling, which can impact farmers’ health. One farmer described an event that occurred during the planting season:

During the nugan [planting], one, someone falls down, because it is steep like this. (DR, Male, Penan, 50, VI)

Furthermore, because of the steep slope, some farmers have difficulty working in rice fields and therefore avoid using steep land for cultivation; however, this is dependent on land availability. Nevertheless, most farmers do not consider steep slopes in a dry rice field to be an obstacle to rice agriculture. Erosion was also mentioned as a consequence of a steep slope and can be associated with poor soil quality. One farmer mentioned that the yield per area was lower in steep slopes since rice plants were planted farther away than in flatter areas. Due to the steep slope problems, some farmers stated as a remedy to choose flat land or avoid using too steep slopes to cultivate the dry rice agroecosystem. The problem is connected to microrelief microzones with slope differences and to the landscape element of a hill as a topographical factor.

#### Vegetation problems

**Fig. 7:**
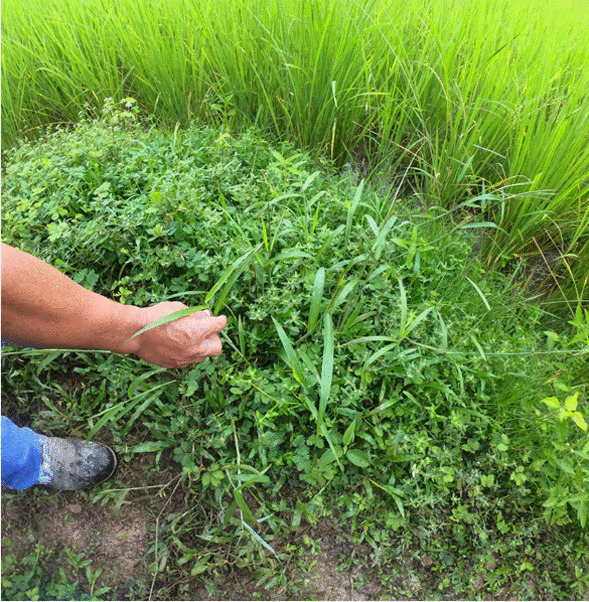
In an interview, a farmer explained that weeds enter the wet rice fields and cause problems for rice plant growth (Photo: A. Hollaus)

The occurrence of weeds (Fig. 7), such as *Imperata cylindrica* and *Dicranopteris linearis,* is a major problem perceived by farmers as affecting rice plant growth through competition for nutrients and sunlight and the spread of the weed over the rice plant, and can lead to poor soil quality. Furthermore, thorny weeds pose a health risk of injuries to farmers during hand-weeding.

The causes of weed occurrences are often connected to other problems in the RAEs. The spread of fire during clearing and erosion create areas to which weeds can migrate. Insufficient water availability and unadjusted water levels, as well as uneven field levels in wet rice fields, lead to dry places where weeds can grow. Furthermore, poor soil quality and a lack of weeding because of insufficient time or farmers’ health issues can be a cause of weed occurrence in rice fields. Weeding is seen as difficult and time-consuming work, but necessary.

You have to clear the paddy [rice field], so the paddy [rice plant] can grow very well. When this type of grass is grown under the paddy, we have to weeding it. (DR, Male, Sa’ban, 61, VI)

Besides hand-weeding, the use of knives, machines, burning or synthetic herbicides are mentioned as forms of weed management. Furthermore, flooding of wet rice fields is regarded as a means of suppressing weeds and therefore needs to be retained. Another method mentioned by farmers for controlling weeds is by maintaining a clearing of vegetation around the field to reduce weed migration. Participants mentioned that some of their solutions to control weeds can at times be ineffective or result in even stronger weed regrowth.

Farmers mentioned weeds growing in patches in rice fields or infesting entire areas of the field. Microzones related to weed problems were connected to patches of poor rice plant growth, weed occurrence and water availability differences, and in wet rice fields also to field level and water level differences. Landscape elements, such as fallow land, garden, hill, rice fields, grassland or bushland, were a concern for rice farmers due to possible weed migration into the rice field. Farmers mentioned avoiding planting rice in weed-dominated areas, e.g. grassland, and waiting until natural succession in the fallow period produced shady areas and outcompeted the weedy plants.

Another vegetation problem mentioned as causing poor rice plant growth was the surrounding vegetation of rice fields, forming shady areas or causing branches to fall onto the rice field and managed by farmers by clearing the vegetation, such as in landscape elements of fallow land, gardens, tree and vegetation patches or hills.

#### Weather problems

Farmers perceived seasonal variations in the weather and stated that the agricultural year in which the interview took place had been poor for rice plant growth, resulting in reduced yields. Planting too early or too late can cause problems due to the absence of the seasonal weather conditions needed for rice growing. Weather problems were related to droughts, but also to excessive rain or hot temperatures.

Kalau dia terlampau panas pun tak bagus, kalau dia hujan selalu pun tak bagus. [If it’s too hot it’s not good, if it’s too much rain it’s also not good.] (DR, Female, Kenyah, 30)

Besides affecting the rice, heavy rain resulted in slippery conditions and hot temperatures could impact farmers’ health. To cope with hot temperatures, landscape elements of tree and vegetation patches and fallow land reduced the problem by offering shade in which farmers can rest. Furthermore, some farmers’ strategy in the event of a poor agricultural year, e.g. because of drought, was to store enough rice from one harvest to last several years.

Another weather problem was strong wind connected to high plant growth and a wet or weak rice stem resulting in lodging. Consequently, rice grains can fall into the water or onto the ground, resulting in moist grains or the grains being eaten by rats. To avoid lodging, farmers suggested reducing the risk by draining the rice field before harvest or using a rope to support the rice plant. Lodging was indicated by the microzone of lodged rice plant patches.

### Social problems

#### Human disturbance

Human disturbance occurs when humans walk through or along the rice field, stepping on and damaging the rice plants and consequently leading to poor rice plant growth. The problem was related to the landscape element of tree and vegetation patches where people harvest plants and cross the rice field to reach these areas. Furthermore, the landscape element of path infrastructure was linked to human disturbance of rice plants on the field edge coming onto the path and people stepping on the plants. To prevent rice plants from growing onto the path, one farmer constructed a fence-like barrier along the field edge.

Fish in rice fields are another reason for human disturbance. Although the fish themselves were not perceived to be a problem, with just one farmer mentioned fish digging into the bunds and causing damage, some people caught the fish in the wet rice field, damaging the rice plants. Apart from the field owner personally catching the fish, no action was taken. Farmers identified the landscape elements of rivers and rice fields as a source of fish migration to the rice field.

#### Hard to afford and lack of access to paid labour, machinery, farm inputs, tools or irrigation infrastructure

Some farmers said that they lacked the financial resources or access required to obtain tools, farm inputs and machinery such as a bulldozer for levelling, the irrigation infrastructure or labour for rice field maintenance. The causes were linked to the farmers’ lack of income or lack of support from the government or non-governmental organisations (NGO). As a consequence, farmers who do not have access to a bulldozer have to level the field manually, resulting in a slower gradual expansion of the wet rice field or cultivation of a smaller field. Paid labour was not common, but a few Sa’ban and one Penan said that they paid people to manage all or some of the tasks associated with cultivating rice fields. In the instance of farm inputs of synthetic pesticides and fertilisers, some farmers noted that the government provided those for free, but others said that they needed to purchase the inputs, which may be problematic for certain farmers. As a result, these farmers could not use pesticides and had to continue to weed by hand. A similar problem was articulated for the irrigation infrastructure, which could result in a poor water supply and lead to the continuing cultivation of dry rice fields rather than wet rice fields. Some Penan farmers indicated that they wanted to have irrigation pipes, but these were hard to access and they had insufficient funds.

If we have a pipe like others. That is the problem. We don’t have enough pipes. We want to make a taka [wet rice field] […]. We don’t have a pipe to take the water, that is the problem. (DR, Male, Penan, 60)

Solutions to this problem were asking for help in the community if the work was difficult, omitting certain tasks in the rice field or just extending the rice field more slowly. Furthermore, some people looked for work to earn money or used the support of the government or NGOs in certain agriculture projects. The problem was connected to the landscape element of irrigation infrastructure as one important source of the water supply.

#### Other tasks/jobs besides farming

Apart from the tasks in the rice fields, some farmers highlighted their need to have other work to earn money to support their families or afford farm inputs, tools or paid labour, reducing the amount of time available in the rice fields.

Because no money when our children go to school, no money. And that’s why we go to Miri to working. And then [earn] money for our kids to [go to] school. (DR/WR, Male, Sa’ban, 53)

There was a conflict for farmers between the need for money and the need to cultivate and manage the rice field. The first author frequently observed that rice fields were not being weeded, indicated by the microzone of weed occurrence. When asked why that was the case, the interpreter explained that the owner might be absent from the village due to employment in the city, for example. Additional employment often led farmers to neglect their activities in the rice fields, e.g. weeding or proper levelling, when there was insufficient time. However, the tasks could be completed later, but the reduced care of the rice field affected rice plant growth.

#### Poor access to rice fields

Poor accessibility to the rice field was related to a very long walk from the village and the condition of the path infrastructure. Farmers reported frequently choosing rice fields near the village to save on travel time and avoid long distances for rice transportation. One farmer did not cultivate his wet rice field owing to its remote location and planted a dry rice field instead that was close to a dirt road and easily accessible by vehicle or motorbike. Thus, if farmers had no land near the village, some mentioned borrowing land to have the rice field closer. Particularly for farmers in poor health, a nearby rice field was indicated as preferable.

The kind of path infrastructure (e.g. walking path or dirt road) played an important role in how to access the rice field, allowing the use of vehicles for travel and transportation. The poor condition of the path infrastructure was also an issue, making the path inaccessible for motorbikes or cars.

The landscape elements of path infrastructure and village played an important role in the rice field’s accessibility and was critical in selecting the site of rice fields, particularly dry rice fields. Rivers were still used to access rice fields and transport rice yields by boat, especially in Long Lamai where there are no roads.

#### Poor capability of farmers

The capability of a farmer to carry out difficult work in the rice field was often linked to the farmer’s age and health. For example, elderly farmers in poor health had difficulty accessing remote rice fields or undertaking field tasks such as field preparation or weeding. Consequently, they wanted to acquire a rice field near the village or obtain help from others, even paying them. One participant stopped dry rice farming because of her advanced age and focused only on the wet rice field. Her children took over the cultivation of the dry rice field and also assisted with difficult tasks in the wet rice field. The problem was connected to the landscape element of village and its distance from the field.

#### Taboos

Generally, farmers stated that old taboos no longer had any power or influenced their rice cultivation practices. Nowadays they believe in God and are Christian. However, one sociocultural landscape element and natural ecosystem were related to taboos by two farmers in Long Banga: ancient graves and salt springs where rice fields should not be established. This related to the notion that spirits or ghosts inhabit the old stone graves and salt springs, and if disturbed farmers could be afflicted by sickness. Farmers addressed this problem by avoiding spiritually significant areas.

### Agricultural technology system problems

#### Spread of fire

Farmers reported problems of fire accidentally spreading during the burning process in a dry rice field, burning a tree or a larger area than anticipated.

During the burning season, then it is often. People don’t follow the [right] time. This is why it happened. […] Usually, if [it is] a fruit tree, we don’t want to cut it, but mostly it wouldn’t [be] saved because of the fire. (DR/WR, Male, Penan, 70)

This can result in the intrusion of weedy plants into the burnt area and rice field. A further consequence can be that the fire destroys trees and areas that are valuable for various uses (e.g. fruit trees). Therefore, landscape elements connected to the problem were tree and vegetation patches that are accidentally burnt. One Penan farmer explained that to keep the fire under control, all the neighbours of the field should be notified and the field should be burnt when the sun is not as intense and the wind direction is favourable.

#### Insufficient water availability

The problem of insufficient water availability resulted in poor rice plant growth and in wet rice fields led to the occurrence of weeds and hard soil conditions. One Penan farmer described plots in his wet rice field as *"mulah mapeu"*, referring to dry plots with dead rice plants, and led to these plots being abandoned. Insufficient water availability could be caused by dry weather conditions since most fields relied on rain for water. Therefore, planting at the appropriate time was important:

[In the wet rice field], if you plant in the dry season the paddy will die. It [...] needs water. […] if you drop the seed into the soil it needs to sit with water. You plant it if no rain, it will […] die. Seed will not grow. Hill paddy [dry rice field] [..] also needs water and rain to grow. (DR/WR, Male, Sa’ban, 60)

Another cause of insufficient water availability was an unevenly levelled wet rice field. For example, one farmer mentioned a problem where water was flowing to one side of a poorly levelled field, indicated by healthy growing plants on that side of the field and poor growing plants on the other. Another cause mentioned by farmers was the absence of an irrigation infrastructure, such as pipes, or a broken infrastructure, making the wet rice field more dependent on rain. Only a few areas in both villages had an established irrigation system, such as in Long Banga, where a pipe transports water to the rice field and then flows through the following rice fields. As the farmers explained, one advantage of pipes over other water sources was the ability to regulate the water supply to the field and thus adjust the water level. However, poor irrigation arrangements among pipe users or the blocking of the water flow through the rice fields by one of the field owners could also lead to insufficient water availability. In Long Lamai, for example, a pipe delivers water to several wet rice fields, and fields at the far end of the pipe struggle to get water if the outtake upstream is too high. One farmer solved this problem by using excess water from the neighbours’ rice field instead of the pipe. Other solutions were to grow dry rice cultivars in wet rice fields, apply weed management, re-level the field, maintain the irrigation infrastructure or abandon the wet rice field or plot. If no water was available for a wet rice field, farmers continued to cultivate dry rice fields. For dry rice fields, some farmers mentioned that maintaining a vegetation buffer space above the field should serve as a water retention area.

Microzones associated with insufficient water availability were soil texture differences, field level differences and water level differences in wet rice fields. In the dry rice field, one farmer highlighted that water availability was better down the slope than up it, and was therefore associated with slope differences. The problem could lead to patches of poor rice plant growth and yield differences in the field.

The landscape elements that contributed to insufficient water availability were grassland above rice fields, due to poor water retention, and hills, which were mentioned as having less water availability than areas in the valley. Rivers were also mentioned as producing sediment and thus blocking the irrigation pipe. Additionally, other rice fields and irrigation infrastructure were linked with the problem by the mismanagement or lack of irrigation infrastructure. Fallow land, fishponds, rivers, irrigation infrastructure, other rice fields and springs as sources of water were all linked to water availability and hence were mitigating the problem.

#### Lack of suitable land

A lack of suitable land was frequently mentioned as a problem by farmers as having an impact on the field’s location and the quality of the land used. If no suitable land was available, the solutions were to use the land that is available or to borrow land. With land that was borrowed, the owner had the option to tolerate but also to restrict its use, which was connected to a limitation on producing long-term crops such as fruit trees or coffee plants. The borrowing of land for rice cultivation was repeatedly mentioned by the Kenyah in Long Banga.

Actually, this is not our land. We just borrow [it], just for this year. (DR, Female, Kenyah, 30)

Land ownership was generally acquired by cultivating an unused area. To keep the land, participants took measures to assure ownership to limit the danger of land claims that could result in land conflicts. As a result, farmers returned to the land for rice cultivation or tree planting to retain ownership of the land.

Don’t let it [the land] be like this, they come slashing and they take your land for plant paddy [rice] or everything. If a long, long time didn’t come and check it, they take it long, long time; three, four, five years, then all is gone. (DR, Male, Penan, 60)

For wet rice fields, if no suitable land was available, farmers needed to continue to cultivate dry rice fields. Land availability for wet rice fields was seen as difficult since the field type needed *“flat land and the water” (Sa’ban, 65, Male, WR),* while interviewees saw almost no restrictions on making a dry rice field, as one participant commented: *“[…] we can do it everywhere.” (DR, Female, Sa’ban, 57).* One Sa’ban remarked on the difficulties of getting flat land for the wet rice field in Long Banga:

The Kenyah people they plant it because they have no land, so they plant the paddy [wet rice field] in the parit [street trench]. (WR, Male, Sa’ban, 65)

Land selected by farmers for a new rice field was assessed for soil quality by plant and soil indicators. To address the problem of poor soil quality, farmers stated that such sites are avoided during land selection. Landscape elements connected to the lack of suitable land were fallow land and its age as an important factor for the soil quality in dry rice fields. Furthermore, the valley areas were important for establishing a wet rice field and farmers tried not to use weed-dominated areas in bushland or grassland, reducing the possible area to make a rice field.

#### Insufficient rice yield

Another, infrequently mentioned problem was the insufficiency of rice yield for the farmer’s needs. For most respondents, their rice fields provided enough rice to meet their demands. Some even had enough rice to sell the surplus. However, one farmer said that for Long Lamai:

Now some people no paddy [rice] [..] to eat. Finish... (WR, Male, Penan, 57)

He continued by explaining that they would go to work to purchase the rice. However, farmers acknowledged that they helped each other and shared rice with others who were in need. To have enough rice, one strategy explained by a Kenyah farmer was to plant rice in sufficient quantities to store it for two years in the event of a poor harvest one year. Another strategy was to plant both rice field types to minimise the risk of a poor yield due to the failure of only one RAE.

They need both. Because they don’t know if taka [wet rice field] will live good or the terek [dry rice field] is much better... (WR, Female, Penan, 27, VI)

#### Poor soil quality

One problem mentioned by many farmers was poor soil quality in the rice field, resulting in weed occurrence, poor rice plant growth and a low yield. Poor soil quality also affected the usage frequency of dry rice fields on the same land area. Farmers mainly used the properties of soil colour, texture and hardness to describe soil quality. Farmers mentioned two dominant soil colours, black and red, sometimes also labelled as yellow instead of red. According to the interviews, the red soil is underneath the black soil.

Black soil is on the top on the surface of the earth and under there red soil. (DR, Male, Kenyah, 62, VI)

While the red soil was described as infertile, the black soil was perceived to be fertile and should be used for rice growing. Soil hardness was related to hard and soft soil. Hard soil is dry and provides poor water availability for rice plant growth. Farmers noted that hard soil was difficult for the rice plant roots to penetrate and caused difficulties during direct planting with a stick. In contrast, soft soil promoted rice plant growth and healthy green rice plants. Soil texture was perceived to vary between clayey, sandy or stony soils—one farmer even added loam—all of which have different effects on rice plant growth, soil moisture and water retention.

The perceived causes of poor soil quality were a short fallow period, overuse of the soil and poor fallow vegetation, insufficient water availability to make the soil soft or the topographic location of the rice field, with better soil conditions in the valley than on hills. A cause of poor soil quality in wet rice fields was poor levelling of the pond by removing the fertile black soil until the red soil appeared, especially if machines (bulldozers) were used:

Machine [for levelling] is not good for this wet paddy field. (DR, Male, Sa’ban, 58)

Another cause that farmers with dry rice fields mentioned was soil erosion, which can result in the displacement of fertile soil.

Solutions to mitigate poor soil quality were to relocate (shift) the dry rice field, check for suitable soil during land selection and longer fallow periods. A farmer said that he had reduced the size of the rice field that year since the soil quality in one section was poor, and he was just using the fertile part of the field. Thus, before moving the dry rice field to a new location, farmers decide whether another year of cultivation is possible:

If it is subur [fertile] then we will plant on it [the land], but [if] not, then we can change to others. (DR, Male, Kenyah, 59, VI)

However, the number of possible cultivations was limited by the soil’s quality and previous land cover; for example, if a farmer wanted to use grassland or bushland for a rice field, the field would be useful, according to one farmer, for no more than one cultivation cycle. Some farmers coped with poor soil quality by applying synthetic fertiliser or said that they kept a vegetation strip above the field. In wet rice fields, water was maintained to keep the soil soft, and if red soil appeared during levelling, farmers put fertile black soil back on top.

Microzones connected to poor soil quality were soil colour, soil hardness and soil texture differences. Besides using soil properties to determine soil quality, farmers also used plants as indicators. The yellow colour of the rice plant, for example, indicated poor soil quality.

This is the tanah [soil], that not good. You will see the paddy [rice plant] yellow and the soil is yellow. That is the proof that the paddy not good. (DR, Male, Kenyah, 59, VI)

One Penan farmer assessed soil quality by the appearance and type of plants found in an area.

This is Ureu Kemanen [type of grass], […], you see there many, […], if this grows many like this, you are slashing here, you grow the paddy: good. (DR, Male, Penan, 60)

A Sa’ban farmer used the soil properties as important indicators of land quality, which needed to be verified before selecting land:

We are looking for the, for the soil first. If the soil is black, the paddy is good. (DR/WR, Male, Sa’ban, 60)

However, yield differences and slope differences were also linked to soil quality. Furthermore, larger rocks in the fields were recognised as a lost area for rice planting.

The landscape element related to soil quality was fallow land, which allows the area to recover and improve soil quality. Farmers also associated primary forests with fertile soil. Furthermore, one farmer reported differences in soil quality between hill and valley areas. Valleys were thought to have better soil and to be cooler since they were closer to the river than hills, where stony soil can be found. Grassland and bushland with a dominance of weeds were also avoided because of the poor soil quality they indicated, such as hard conditions. Rivers were reported to cause erosion after heavy rain by displacing soil from the rice field or sediment onto the field. The same was mentioned by one farmer for the road above his rice field where soil run-off relocated poor quality soil to his field.

#### Synthetic fertiliser application

The problem mentioned by farmers in relation to synthetic fertiliser was its application. Synthetic fertilisation, according to farmers, was more harmful to the rice plant and its quality than it was beneficial, as one Sa’ban explained for avoiding synthetic fertiliser:

Because the baja [synthetic fertiliser] […] the quality of the paddy is not good. Because when the paddy [rice plant] is too big, the paddy cannot stand. It will fall down. (WR, Female, Sa’ban, 24)

In comparison with pesticides, synthetic fertiliser application had so far been quite infrequent in both villages, as farmers perceived their land to be sufficiently fertile.

Also, if the paddy [rice plant] is good, no need to use the baja [fertiliser], only need to spray [pesticides], that’s all, to kill the weed. We seldom use the baja, the fertiliser. (DR/WR, Male, Sa’ban, 60)

In Long Lamai, only one farmer said that he used synthetic fertiliser. Nevertheless, other farmers in Long Banga said that they used synthetic fertiliser only if the soil was poor quality. Although synthetic fertiliser was associated with increased rice plant growth, according to farmers it came at the expense of low seed quality and the possibility of rice plants lodging, which was related to the microzone of lodged rice plant patches. Farmers were thus trying to avoid using synthetic fertiliser. In the wet rice field, several farmers mentioned that before planting they simply chopped the grass and let it decay like a natural fertiliser. Vegetation above a rice field was considered by some farmers to be an additional source of natural fertiliser for the rice field.

#### Synthetic pesticide application

Synthetic pesticides were applied to control pests, but were mostly used to control weeds in rice fields, especially in dry rice fields, where farmers frequently said herbicides were used. Pesticides were applied to wet rice fields before planting and flooding, or only to the bunds.

At the terek [dry rice field], sometimes they apply the spray […] at the place there is ureu [grass], but at the taka [wet rice field], they spray the bunds (DR, Male, Penan, 50, VI)

Farmers expressed concerns about the use of pesticides, which could harm the health of the rice plants. Furthermore, they explained that certain weeds grew again or were resistant to herbicides. Pesticides, according to the respondents, caused health risks and could pollute water. Another consideration was that rice that had been treated with pesticides could not achieve a higher selling price. As a result, some farmers tried to avoid pesticide application and continued to perform weeding. Pesticide management was related to the microzones of weed occurrence, soil quality differences as well as poor rice plant growth patches, and to the landscape elements of rivers and fishponds due to the ensuing pollution of water.

#### Unadjusted water level

If the water level is too high, rice plants will be susceptible to lodging and, especially in the early growing stage, will drown. However, if the water level is too low this can encourage weed occurrence. One example was given by a farmer who had to lower the water level to eradicate the golden apple snail in one of his wet rice field plots, which affected rice plant growth. Furthermore, a high level of water in the wet rice fields makes harvesting difficult because of the issue of moving around and the danger of dropping the panicles into the water. Before the harvest, farmers blow the water out of the wet rice field, allowing them to move easily through it. This problem was connected to microzones of lodged rice plant patches and water level differences.

## Discussion

In this study, we identified the problems, causes and consequences of problems as perceived by farmers, as well as the solutions applied in the local rice field agroecosystems (RAEs) in the upper Baram, Sarawak. Since ecological processes can take place across different spatial scales [72, 73], our study provided a broader view of perceived problems in RAEs by using the surrounding landscape elements and microzones in the rice fields as analytical units. Our study demonstrated that indigenous farmers in the research area had important information about the problems and spatial relations associated with RAEs.

The findings indicated that the indigenous farmers in Long Lamai and Long Banga not only perceived environmental problems in the RAEs, but social and agricultural technology system problems as well, often with several complex and interrelated causes. For example, insufficient water availability, mainly an agricultural technology system problem in the wet rice fields, could have underlying environmental causes, e.g. droughts, or social causes, e.g. poor irrigation organisation. In rainfed dry rice fields, the problem was mostly an environmental issue related to rain conditions. Therefore, the magnitude and severity of the problems could vary between the different RAEs. While water-related problems were major concerns in wet rice fields, farmers perceived the occurrence of weeds to be more problematic in dry rice fields. Farmers could also perceive a problem on different spatial scales. For example, insufficient water availability was connected to landscape elements, e.g. irrigation infrastructure and rivers, which supply water, or on smaller scales to microzones, such as water availability differences or water level differences in the rice fields. Furthermore, while certain problems influenced the RAEs directly, some problems led to other issues, forming a causal chain. Cramb et al. (2009) describe such causal chains by the abandonment of the shifting cultivation fields of one household, which leads to a higher pest risk for the remaining fields, which again results in a higher workload for the remaining farmer to control the pests, but also to maintaining the RAE with fewer people in the labour-exchange group, which consequently impacts their motivation to continue shifting cultivation, leading to a cycle of abandonment of rice fields [14].

Many of the problems related in the interviews, including animal disturbance, insufficient water availability, poor soil quality, vegetation problems (e.g. weed occurrence), weather problems (e.g. droughts), human disturbance and synthetic pesticide application, were perceived to have a direct impact on agricultural productivity due to their impact on rice plant growth. Similar perceptions about the negative effects on rice production have also been noticed by Thai farmers in northern Vietnam due to irrigation water scarcity [74] or in Banaue, Philippines, as a result of native and invasive pests [12]. Furthermore, in Tanzania farmers have noted animal disturbance, droughts and poor soil fertility [75] and in Rwanda water shortages, lack of inputs, rice diseases and soil fertility decline as major constraints in rice productivity [76].

In dealing with such concerns, farmers apply a range of problem-solving strategies, either before (preventive) or after (reactive) the problem has occurred. Preventive strategies aim to mitigate or influence the problem before it occurs, such as avoiding unsuitable areas for rice cultivation or spreading the risks of pests through social agreements on community planting times. Growing both wet and dry rice fields was seen as a precautionary step in case one type fails. Another preventative method was to ensure the storage of enough yield for at least two years in case there is a poor agricultural year, e.g. due to weather problems, and a poor rice harvest. Similarly, Hosen et al. (2020) state in their research, including Long Lamai and Long Banga, that farmers store rice in addition to other traditional adaptation strategies such as shifting agriculture, intercropping or forest preservation in order to cope with climate change issues [40].

Reactive solutions are intended to address the cause of an existing problem or to alleviate or mitigate the severity of its consequences, such as weeding or the use of pesticides for insect problems. In response to certain problems, farmers could abandon their rice fields, for example fields with poor accessibility that makes the journey to the rice field unattractive. Like farmers in Rumah Ranggong, Sarawak, the farmers in the research area preferred field locations next to logging roads for easier access [30]. Poor accessibility was also a major reason behind the local people in the Shexian Dryland Terrace System abandoning their fields [77].

The problem-solving strategies in the villages were firstly based on indigenous farmers’ traditional knowledge about their landscape and rice farming. For example, shifting cultivation is used to manage dry rice fields, which requires farmers to have an understanding of soil processes to determine when poor soil quality requires them to relocate the field, to know how the soil recovers best during the fallow period and how to identify a good soil quality [78]. In the research area, the farmers used their knowledge about weather phenomena to identify the right burning and planting time. Also, the Kenyah Badeng in Sarawak use their traditional knowledge to predict weather conditions to plan activities in rice fields [79]. However, the traditional knowledge also helped to deal with problems and disturbances. Hosen et al. (2020) found for Long Lamai and Long Banga that the traditional strategies for coping with environmental disturbances and uncertainties in the villages are based on adaptive management and aim to maintain ecosystem and community resilience [40]. Therefore, indigenous farmers are reacting and adapting to disturbance based on their traditional knowledge, which supports the resilience of farmers to problems and change [11]. An interesting case is the former nomadic Penan in Long Lamai, but the same is true of other Penan groups, who only started to cultivate rice shortly after they settled in the 1950s, and farming practices were introduced to them by missionaries and neighbouring ethnic groups who are traditional agriculturalists [59, 80–82]. Therefore, even though the Penan already had profound knowledge of the surrounding landscape and forest resources [81, 83], compared to the other ethnic groups, farming is quite new to them and their culture. Thus, a substantial amount of the Penan’s knowledge related to rice farming is originally based on the traditional knowledge of other ethnic groups, but has now integrated the Penan’s own experiences.

Moreover, new emerging problems in the RAEs, such as invasive weeds or the introduction of the golden apple snail, required farmers to find new problem-solving techniques, which often led indigenous farmers to integrate scientific knowledge and industrial agricultural technologies, such as the use of synthetic pesticides, in their RAE management. The use of different knowledge systems can be found also for other indigenous or local groups, which leads to hybridising indigenous and scientific knowledge [21]. Dawoe et al. (2012) demonstrate that farmers in Ghana’s Ashanti area build their soil fertility strategies on a hybrid knowledge system by integrating traditional knowledge with new ideas from outside that can replace the old traditional methods [84]. The adoption and integration of scientific knowledge systems into local agricultural practices can have a positive impact on biodiversity and ecosystem services [20, 85]. However, through the integration of modern farming practices, the traditional knowledge systems may be eroded, with remnants of this traditional knowledge only maintained by the older generations [86].

Furthermore, the uptake of industrial agricultural technology might create new problems, some of which are also social in nature. In our research, the limited affordability of farming tools, inputs or labour is an example of the problems related to industrial agricultural technologies. The consequences of such problems are shown for the coastal region of Sarawak, where rice farmers face difficulties with buying farm inputs and machinery that affect their incomes and lead to poverty and migration to urban areas [34]. Other consequences can be that farmers are unaware of the impact of industrial agricultural technologies. Amster (2008) reported that only a few Kelabit, in the Kelabit Highlands of Sarawak, were aware of the hazardous threat posed by the pesticides frequently applied in wet rice fields [35]. As observed in the rice terraces in Banaue, Philippines, a change in traditional practices, such as not following the community planting time, can lead to a rise in pest problems [12]. In another study about the Ifugao in Banaue, the shift from traditional organic to inorganic agricultural methods was perceived to have a detrimental impact on soil and water [22]. Furthermore, agricultural intensification can have a negative influence on the landscape and ecosystem services, such as pollination and biological pest control, which might lead to additional costs for farmers to replace these services [73, 87, 88]. For example, farmers in Cai Be, in Vietnam’s Mekong delta, are aware that the use of pesticides in the RAE affects the surrounding areas and also drinking water quality [24].

Agricultural activities are not only perceived to have an impact on the surrounding landscape; our study also demonstrated that indigenous farmers perceived problems in the RAEs to be caused by surrounding landscape elements. For example, animal disturbance and weed occurrence were perceived to be problems that were rooted in surrounding landscape elements such as fallow land or grassland, since they were perceived to serve as habitats for pests and weedy plants. Two indigenous farmers in Long Banga also believed that disturbing landscape elements such as salt springs or ancient graves by agricultural activities could cause diseases for farmers and thus should be avoided when selecting land for a rice field. A similar former belief was demonstrated by the neighbouring Kelabit who were not allowed to enter salt lick ecosystems owing to fears of becoming sick [35]. Problems for the RAEs caused by the surrounding landscape elements are also shown in the rice terraces of Ifugao, Philippines, where the adjacent woodlots, called *muyong*, supply water to the rice field, but due to a change in the *muyong* management system, the RAE faces water availability issues [13]. Neighbouring rice fields can also cause problems of weed migration, as perceived by the indigenous farmers in Long Lamai and Long Banga. Farmers in Vietnam’s Mekong delta have observed a similar effect on rice-fish fields caused by pesticide-polluted water from adjacent rice fields, which has a detrimental effect on the fish and requires farmers to stop water inflow or maintain a buffer field around the rice-fish field, or even prevent farmers from starting rice-fish fields [24]. However, the landscape elements, besides being a source of problems, were also perceived to have a function in the mitigation of problems. For instance, path infrastructure was perceived by indigenous farmers to allow a human presence close to the rice fields to reduce animal disturbance in the rice fields. Similarly, local women in Coatitilán, Mexico, perceive that forests and trees prevent soil erosion and that vegetation plays an important role in soil water retention and landscape structure maintenance, thereby protecting agricultural land and productivity [89]. For the mitigation of problems and to compensate for ecosystem service losses, a diverse complex landscape with natural habitats [88] can benefit farmers, such as in a form of pest control [44], or by receiving water for the rice fields from woodlots [12].

At the sub-field level, we found that farmers did not perceive rice fields to be homogeneous spatial unit, but rather spatially varying microzones. The Tsimane’ in Bolivia recognise such sub-unit patches mostly by the dominance of plant species and only one kind of patch based on soil type [16]. The farmers in the upper Baram described microzones based on biotic and abiotic criteria, and used these spatial sub-units as indicators to deduce underlying problems, such as animal disturbance, weed occurrence, insufficient water availability or poor soil quality. Indicators in the rice fields are critical for making management decisions and applying solutions to problems. For instance, a rice plant’s appearance, such as microzones formed by the rice plant colour, was a key indicator of potential problems. Another important microzone indicator was the soil colour to assess the soil quality. The use of soil and plant indicators has also been documented for other ethnic groups on Borneo, as Siahaya et al. (2016) describe for the Dayak Tunjung farmers in East Borneo who use plant species indicators to monitor soil quality for rice cultivation on an annual basis and to decide whether the field need to be abandoned [78]. Also, outside Borneo, soil and plant indicators are used to assess the quality of soil, for example in Brazil [90] or Laos [91]. Farmers in Ghana’s Ashanti area regard soil colour, crop yield, water-holding capacity, foliar colour and weed occurrence as important indicators of soil fertility [84]. These indicators, along with microzones of soil texture, soil hardness and rice plant growth, were also observed in our study to evaluate the quality of the land. Farmers noted that they adapted their management to some microzone properties, for example manual weeding by pulling was only essential in weed-infested microzones of the rice field. In the Dindori district of Madhya Pradesh, Central India, indigenous farmers also perceive micro-farming situations by different soil colours, topography, crop, irrigation source and overall problems and plant native rice varieties by following location-specific strategies [92].

## Conclusions

This study examined indigenous farmers’ perceptions of problems in local rice field agroecosystems (RAEs), their causes and consequences, and the solutions applied, and how these problems were connected to surrounding landscape elements and microzones. The use of qualitative oral interviews in combination with visual sketch drawings facilitated interviews in a multilingual context and aided with identifying landscape elements and microzones and their relationships with the RAEs.

The findings of the study showed that indigenous farmers associated problems with landscape elements and microzones, indicating the indigenous farmers’ thorough understanding of the functioning and connectivity of spatial units at various spatial scales. This understanding, which was rooted in farmers’ traditional knowledge of their environment, served as the framework for dealing with problems and disturbances. However, as demonstrated, indigenous farmers often integrated scientific knowledge and industrial agricultural technology to deal with the problems they encountered. The hybridisation of knowledge systems was the consequence of this adoption of industrial agricultural practices, which comes with a loss of traditional knowledge and might change the sociocultural life of indigenous farmers.

Our insight contributes to a better understanding of how indigenous farmers perceive, cope with and adapt to problems in the landscape, which is important for landscape and resource management. By incorporating environmental, social and agricultural technology system problems and different spatial scales, a broader perspective is presented that extends beyond the evaluation of a single dimension of problems and scales. Incorporating multiple dimensions in the research demonstrates that the investigation of just one dimension can result in a dilution of the importance of the landscape for indigenous people and in a failure to reflect its true value [16]. Future ethnoecological landscape research should place a greater emphasis on the challenges in landscapes faced by indigenous people by incorporating multiple scales and dimensions in studies. Additionally, we suggest that local problem solving needs to be supported to ensure that indigenous farmers maintain and continue to benefit from their RAEs. This may be achieved by conducting participatory workshops to identify locally preferred solutions, support the development and implementation of the solutions and upscale successful local solutions to other areas.

## Supplementary Information

**Additional file 1:** Landscape element categories. Categorisation of 44 features included in the sketch drawings and oral interviews around RAEs into 16 landscape elements. The table shows the inductively categorised landscape elements (left) based on the coded features from the sketch drawings and oral interviews (right).

## Data Availability

The tables and text of the article contain the data that support the results. The datasets used and analysed during the current study are available from the corresponding author on reasonable request.

## Abbreviations

RAE: Rice field agroecosystem
WR: Wet rice field
DR: Dry rice field

## Footnotes

1 Estimations are 750 Sa’ban, of whom 350 are in the village and 400 outside it, and 450 Kenyah, of whom 230 are in the village and 220 outside it.
