# Supplementary material for: Indigenous farmers’ perceptions of problems in the rice field agroecosystems in the upper Baram, Malaysia
Source: J Ethnobiol Ethnomed. 2022 Mar 29;18:26. doi: 10.1186/s13002-022-00511-1 (PMC8962147; doi:10.1186/s13002-022-00511-1)
Supplement: Supplementary file 1 — Additional file 1: Landscape element categories. Categorisation of 44 features included in the sketch drawings and oral interviews around RAEs into 16 landscape elements. The table shows the inductively categorised landscape elements (left) based on the coded features from the sketch drawings and oral interviews (right). [file 13002_2022_511_MOESM1_ESM.docx]

| Additional file 1 | | | | | |
| --- | --- | --- | --- | --- | --- |
| File name: Additional file 1 | | | | | |
| File format: .docx | | | | | |
| Title of data: Landscape element categories | | | | | |
| Description of data: Categorisation of 44 features included in the sketch drawings and oral interviews around RAEs into 16 landscape elements. The table shows the inductively categorised landscape elements (left) based on the coded features from the sketch drawings and oral interviews (right). | | | | | |
|  |  |  |  |  |  |
| **Landscape elements** | **List of features around the rice field found in the sketch drawings and oral interviews** | | | | |
| **Topography: Hill and valley** | Hill | Valley |  |  |  |
| **Primary forest** | Primary forest | Native land |  |  |  |
| **Fallow land** | Fallow land | Unplanted area |  |  |  |
| **Bushland** | Bushland | Dicranopterix linearis area |  |  |  |
| **Grassland** | Grassland | Imperata cylindrica area |  |  |  |
| **Tree and vegetation patches** | Tree and tree patches | Water spinach patch | Burnt land |  |  |
| **Rice fields** | Wet rice field | Dry rice field | Nursery |  |  |
| **Garden** | Garden | Tapioca garden | Coffee garden |  |  |
| **Fishpond** | Fishpond |  |  |  |  |
| **River** | River | Riverbank |  |  |  |
| **Spring** | Spring | Salt spring |  |  |  |
| **Path infrastructure** | Path | Bridge |  |  |  |
| **Irrigation infrastructure** | Irrigation pipe and dam | Mini-hydropower plant | Water tank |  |  |
| **Hut** | Hut | Rice bag | Sambar deer cage | Trashing tower | Storage hut area |
| **Village** | Village | House | Airfield | Toilet | School |
| **Sociocultural element** | Ancient graves | Border | Village border | Historical place of village | Other people’s land |
